# 4-[(*E*)-3-(4-Methyl­phen­yl)-3-oxoprop-1-en-1-yl]benzo­nitrile

**DOI:** 10.1107/S2414314620008007

**Published:** 2020-06-26

**Authors:** Dandavathi Arunkumar, Seranthimata Samshuddin, Mhammed Ansar, Joel T. Mague, Youssef Ramli

**Affiliations:** a Department of Chemistry, Sri Dharmasthala Manjunatheshwara Institute of Technology, Ujire -574 240, and affiliated to, Visvesvaraya Technological University, Belagavi, Karnataka, India; b Laboratory of Medicinal Chemistry, Drug Sciences Research Center, Faculty of Medicine and Pharmacy, Mohammed V University, Rabat, Morocco; c Department of Chemistry, Tulane University, New Orleans, LA 70118, USA; Katholieke Universiteit Leuven, Belgium

**Keywords:** crystal structure, olefin, benzo­nitrile, carbon­yl, chalcone

## Abstract

In the title mol­ecule C_17_H_13_NO, the phenyl rings are inclined to one another by 48.04 (9)°. In the crystal, weak C—H⋯π(ring) inter­actions form a layered structure parallel to the *ab* plane.

## Abstract 2

In the title mol­ecule, the phenyl rings areinclined to one another by 48.04 (9)°. In the crystal, weak C—H⋯π(ring) inter­actions form a layered structure.

## Graphical abstract

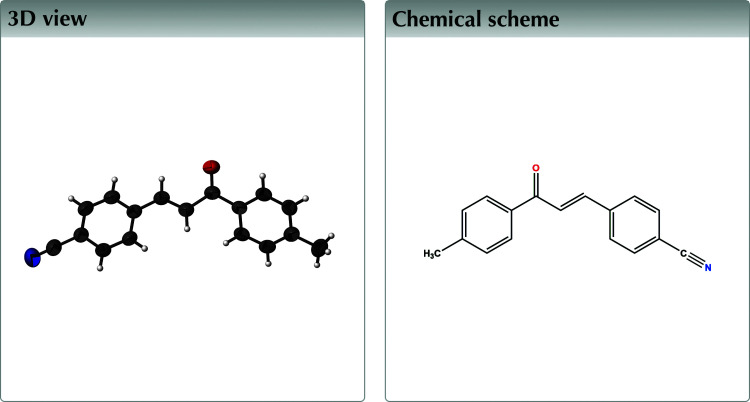

## Structure description

**Figure 1:**
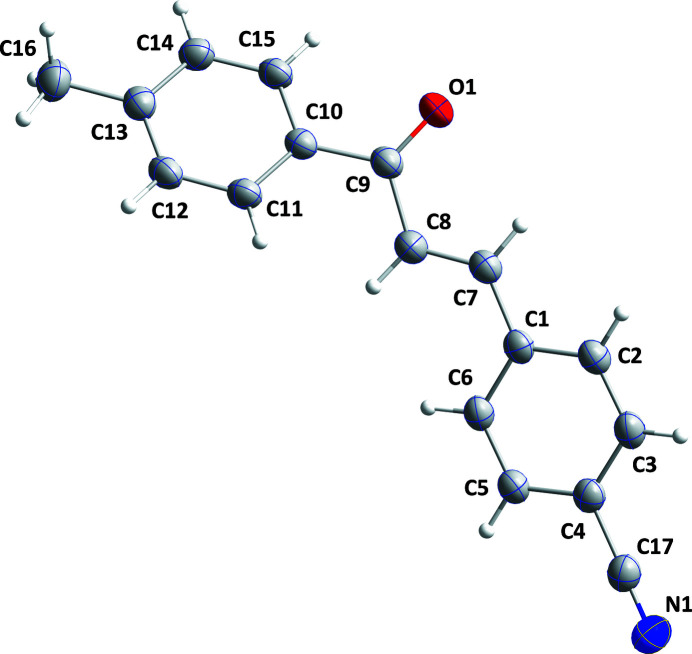
The title mol­ecule showing 30% probability ellipsoids.

Chalcones are compounds that can be easily synthesized, and their analogs can also be isolated from natural products (Dhar, 1981 ▸). Apart from their biological applications, some chalcones with appropriate substituents are also reported to be good NLO materials (Shettigar *et al.*, 2006 ▸). As part of our work in this area, we now describe the synthesis and structure of the title compound (Fig. 1 ▸).

The 4-cyano­phenyl and 4-methyl­benzoyl units are disposed in a *trans* fashion about the C7=C8 double bond. The dihedral angle between the planes of the C1–C6 and C10–C15 benzene rings is 48.04 (9)° and these benzene rings are inclined to the plane defined by the propene atoms C7, C8 and C9 by 16.0 (1) and 32.6 (1)°, respectively, while O1 lies 0.24 (1) Å away from the propene plane.

**Figure 2:**
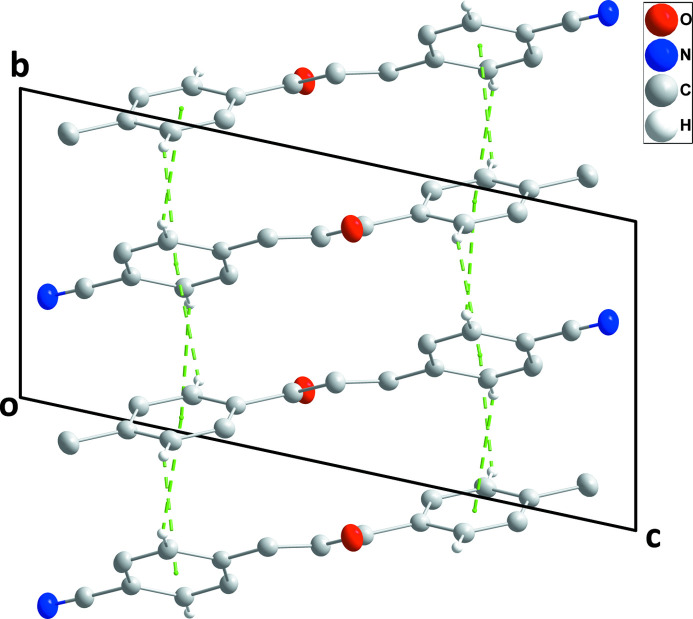
Elevation view of a portion of one layer viewed along the *a*-axis direction with C—H⋯π(ring) inter­actions depicted by dashed lines.

In the crystal, stacked mol­ecules form layers parallel to the *ab* plane with the *para* substituents on the phenyl rings on the outside surfaces of the layers (Figs. 2 ▸ and 3 ▸). The mol­ecules constituting each layer are associated through very weak C2—H2⋯*Cg*2, C5—H5⋯*Cg*2, C12—H12⋯*Cg*1 and C15—H15⋯*Cg*1 inter­actions across centers of symmetry (Table 1 ▸; *Cg*1 and *Cg*2 are the centroids of rings C1–C6 and C10–C15, respectively).

## Synthesis and crystallization

An equimolar mixture of 4-methyl­aceto­phenone (0.01 mol) and 4-cyano­benzaldehyde (0.01 mol) in ethanol (30 ml) was stirred for 3 h in the presence of NaOH (5 ml, 30%) at 283 K. The crude solid obtained was collected by filtration and dried. It was purified by repeated recrystallization. Thin layer chromatography was used to check the purity of the compound. Single crystals were grown from ethanol solution by slow evaporation, yield 86%, m.p. 415 K.

## Refinement

Crystal data, data collection and structure refinement details are summarized in Table 2 ▸.

## Supplementary Material

Crystal structure: contains datablock(s) global, I. DOI: 10.1107/S2414314620008007/vm4044sup1.cif

Structure factors: contains datablock(s) I. DOI: 10.1107/S2414314620008007/vm4044Isup3.hkl

Supporting information file. DOI: 10.1107/S2414314620008007/vm4044Isup3.cml

CCDC reference: 2009913

Additional supporting information:  crystallographic information; 3D view; checkCIF report

## Figures and Tables

**Figure 3:**
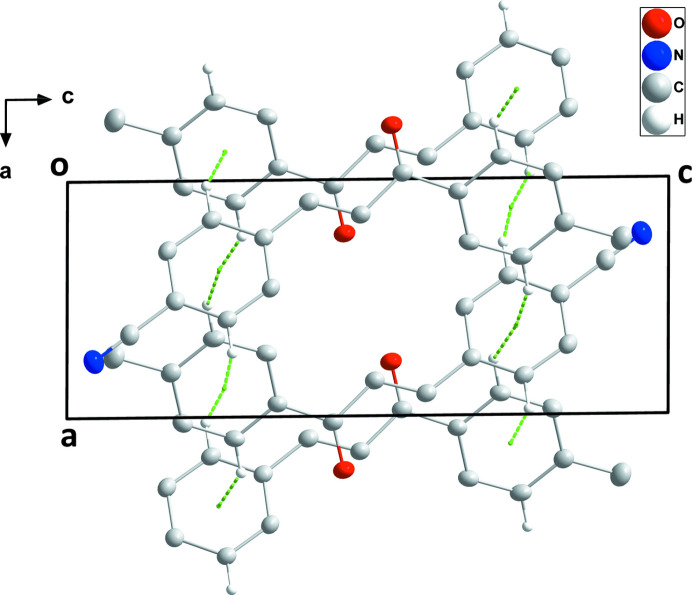
View of a portion of one layer viewed along the *b*-axis direction with C—H⋯π(ring) inter­actions depicted by dashed lines.

**Table 1:** Hydrogen-bond geometry (Å, °) *Cg*1 and *Cg*2 are the centroids of the C1–C6 and C10–C15 benzene rings, respectively.

*D*—H⋯*A*	*D*—H	H⋯*A*	*D*⋯*A*	*D*—H⋯*A*
C2—H2⋯*Cg*2^i^	0.93	2.98	3.645 (2)	129
C5—H5⋯*Cg*2^ii^	0.93	2.91	3.5929 (18)	132
C12—H12⋯*Cg*1^iii^	0.93	2.98	3.637 (2)	129
C15—H15⋯*Cg*1^iv^	0.93	2.99	3.604 (2)	125

Symmetry codes: (i) 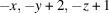 ; (ii) 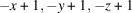 ; (iii) 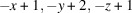 ; (iv) 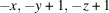 .

**Table 2:** Experimental details

Crystal data	
Chemical formula	C_17_H_13_NO
*M*_r_	247.28
Crystal system, space group	Triclinic, *P* 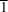
Temperature (K)	296
*a*, *b*, *c* (Å)	5.8686 (2), 7.4955 (3), 15.2792 (5)
α, β, γ (°)	102.195 (2), 90.649 (2), 90.454 (2)
*V* (Å^3^)	656.86 (4)
*Z*	2
Radiation type	Cu *K*α
μ (mm^−1^)	0.61
Crystal size (mm)	0.28 × 0.27 × 0.22

Data collection	
Diffractometer	Bruker D8 VENTURE PHOTON 100 CMOS
Absorption correction	Multi-scan (*SADABS*; Krause *et al.*, 2015 ▸)
*T*_min_, *T*_max_	0.85, 0.88
No. of measured, independent and observed [*I* > 2σ(*I*)] reflections	4923, 2432, 2010
*R*_int_	0.029
(sin θ/λ)_max_ (Å^−1^)	0.618

Refinement	
*R*[*F* ^2^ > 2σ(*F* ^2^)], *wR*(*F* ^2^), *S*	0.059, 0.188, 1.09
No. of reflections	2432
No. of parameters	174
H-atom treatment	H-atom parameters constrained
Δρ_max_, Δρ_min_ (e Å^−3^)	0.21, −0.20

Computer programs: *APEX3* and *SAINT* (Bruker, 2016 ▸), *SAINT* (Bruker, 2016 ▸), *SHELXT/5* (Sheldrick, 2015*a* ▸), *SHELXL2018/3* (Sheldrick, 2015*b* ▸), *DIAMOND* (Brandenburg & Putz, 2012 ▸) and *SHELXTL* (Sheldrick, 2008 ▸).

## full crystallographic data

### Crystal data

**Table:**

C_17_H_13_NO	*Z* = 2
*M**_r_* = 247.28	*F*(000) = 260
Triclinic, *P*1	*D*_x_ = 1.250 Mg m^−^^3^
*a* = 5.8686 (2) Å	Cu *K*α radiation, λ = 1.54178 Å
*b* = 7.4955 (3) Å	Cell parameters from 3797 reflections
*c* = 15.2792 (5) Å	θ = 3.0–72.3°
α = 102.195 (2)°	µ = 0.61 mm^−^^1^
β = 90.649 (2)°	*T* = 296 K
γ = 90.454 (2)°	Block, colourless
*V* = 656.86 (4) Å^3^	0.28 × 0.27 × 0.22 mm

### Data collection

**Table:**

Bruker D8 VENTURE PHOTON 100 CMOS diffractometer	2432 independent reflections
Radiation source: INCOATEC IµS micro–focus source	2010 reflections with *I* > 2σ(*I*)
Mirror monochromator	*R*_int_ = 0.029
ω scans	θ_max_ = 72.4°, θ_min_ = 3.0°
Absorption correction: multi-scan (*SADABS*; Krause *et al.*, 2015)	*h* = −6→7
*T*_min_ = 0.85, *T*_max_ = 0.88	*k* = −8→9
4923 measured reflections	*l* = −17→18

### Refinement

**Table:**

Refinement on *F*^2^	Secondary atom site location: difference Fourier map
Least-squares matrix: full	Hydrogen site location: inferred from neighbouring sites
*R*[*F*^2^ > 2σ(*F*^2^)] = 0.059	H-atom parameters constrained
*wR*(*F*^2^) = 0.188	*w* = 1/[σ^2^(*F*_o_^2^) + (0.1069*P*)^2^ + 0.0967*P*] where *P* = (*F*_o_^2^ + 2*F*_c_^2^)/3
*S* = 1.09	(Δ/σ)_max_ < 0.001
2432 reflections	Δρ_max_ = 0.21 e Å^−^^3^
174 parameters	Δρ_min_ = −0.20 e Å^−^^3^
0 restraints	Extinction correction: *SHELXL 2018/3* (Sheldrick, 2015*b*), Fc^*^=kFc[1+0.001xFc^2^λ^3^/sin(2θ)]^-1/4^
Primary atom site location: dual	Extinction coefficient: 0.045 (6)

### Special details

**Table:**

Geometry. All esds (except the esd in the dihedral angle between two l.s. planes) are estimated using the full covariance matrix. The cell esds are taken into account individually in the estimation of esds in distances, angles and torsion angles; correlations between esds in cell parameters are only used when they are defined by crystal symmetry. An approximate (isotropic) treatment of cell esds is used for estimating esds involving l.s. planes.
Refinement. Refinement of F^2^ against ALL reflections. The weighted R-factor wR and goodness of fit S are based on F^2^, conventional R-factors R are based on F, with F set to zero for negative F^2^. The threshold expression of F^2^ > 2sigma(F^2^) is used only for calculating R-factors(gt) etc. and is not relevant to the choice of reflections for refinement. R-factors based on F^2^ are statistically about twice as large as those based on F, and R- factors based on ALL data will be even larger. H-atoms attached to carbon were placed in calculated positions (C—H = 0.95 - 0.98 Å) and included as riding contributions with isotropic displacement parameters 1.2 - 1.5 times those of the attached atoms.

### Fractional atomic coordinates and isotropic or equivalent isotropic displacement parameters (Å^2^)

**Table:**

	*x*	*y*	*z*	*U*_iso_*/*U*_eq_	
O1	−0.2283 (2)	0.7780 (2)	0.53901 (10)	0.0780 (5)	
N1	0.7607 (4)	0.3451 (3)	0.04455 (13)	0.0857 (6)	
C1	0.2420 (3)	0.6151 (2)	0.32693 (12)	0.0518 (4)	
C2	0.1652 (3)	0.6113 (3)	0.23990 (13)	0.0580 (5)	
H2	0.022952	0.658976	0.231111	0.070*	
C3	0.2945 (3)	0.5390 (3)	0.16681 (12)	0.0613 (5)	
H3	0.240243	0.537500	0.109219	0.074*	
C4	0.5071 (3)	0.4682 (2)	0.17993 (12)	0.0551 (5)	
C5	0.5873 (3)	0.4695 (2)	0.26642 (12)	0.0556 (5)	
H5	0.729197	0.421032	0.274983	0.067*	
C6	0.4562 (3)	0.5427 (3)	0.33882 (12)	0.0558 (5)	
H6	0.510499	0.544070	0.396389	0.067*	
C7	0.0932 (3)	0.6890 (2)	0.40188 (13)	0.0565 (5)	
H7	−0.057548	0.708491	0.387342	0.068*	
C8	0.1490 (3)	0.7312 (3)	0.48794 (13)	0.0606 (5)	
H8	0.299879	0.721005	0.505402	0.073*	
C9	−0.0252 (3)	0.7946 (3)	0.55739 (13)	0.0580 (5)	
C10	0.0541 (3)	0.8750 (2)	0.64974 (12)	0.0519 (4)	
C11	0.2692 (3)	0.9570 (3)	0.66834 (13)	0.0576 (5)	
H11	0.369553	0.959948	0.621986	0.069*	
C12	0.3322 (3)	1.0337 (3)	0.75564 (14)	0.0611 (5)	
H12	0.474772	1.089423	0.766886	0.073*	
C13	0.1902 (3)	1.0302 (2)	0.82681 (13)	0.0587 (5)	
C14	−0.0233 (3)	0.9458 (3)	0.80743 (13)	0.0613 (5)	
H14	−0.121386	0.939348	0.854076	0.074*	
C15	−0.0909 (3)	0.8723 (3)	0.72091 (13)	0.0574 (5)	
H15	−0.235381	0.819966	0.709725	0.069*	
C16	0.2597 (4)	1.1135 (3)	0.92168 (15)	0.0803 (7)	
H16A	0.405925	1.172112	0.922209	0.120*	
H16B	0.268634	1.019697	0.955689	0.120*	
H16C	0.149003	1.201979	0.947754	0.120*	
C17	0.6484 (4)	0.3977 (3)	0.10434 (13)	0.0648 (5)	

### Atomic displacement parameters (Å^2^)

**Table:**

	*U*^11^	*U*^22^	*U*^33^	*U*^12^	*U*^13^	*U*^23^
O1	0.0489 (7)	0.1059 (12)	0.0725 (9)	−0.0032 (7)	−0.0061 (6)	0.0041 (8)
N1	0.0888 (14)	0.1008 (15)	0.0662 (12)	0.0074 (11)	0.0107 (10)	0.0141 (10)
C1	0.0492 (9)	0.0493 (9)	0.0571 (9)	−0.0090 (7)	−0.0044 (7)	0.0121 (7)
C2	0.0506 (9)	0.0646 (11)	0.0614 (10)	−0.0032 (8)	−0.0078 (8)	0.0195 (8)
C3	0.0596 (10)	0.0720 (12)	0.0545 (10)	−0.0105 (9)	−0.0090 (8)	0.0190 (8)
C4	0.0560 (10)	0.0534 (9)	0.0558 (10)	−0.0086 (7)	−0.0011 (7)	0.0115 (7)
C5	0.0489 (9)	0.0591 (10)	0.0593 (10)	−0.0033 (7)	−0.0044 (7)	0.0139 (8)
C6	0.0543 (9)	0.0619 (10)	0.0515 (9)	−0.0046 (8)	−0.0084 (7)	0.0132 (7)
C7	0.0499 (9)	0.0556 (10)	0.0637 (11)	−0.0027 (7)	−0.0052 (8)	0.0120 (8)
C8	0.0503 (9)	0.0696 (12)	0.0606 (10)	0.0019 (8)	−0.0028 (8)	0.0109 (9)
C9	0.0476 (9)	0.0609 (10)	0.0642 (11)	0.0008 (7)	−0.0032 (8)	0.0105 (8)
C10	0.0443 (8)	0.0508 (9)	0.0604 (10)	0.0019 (7)	0.0004 (7)	0.0109 (7)
C11	0.0450 (9)	0.0593 (10)	0.0672 (11)	−0.0029 (7)	0.0050 (7)	0.0105 (8)
C12	0.0457 (8)	0.0578 (10)	0.0758 (12)	−0.0042 (7)	−0.0042 (8)	0.0055 (9)
C13	0.0576 (10)	0.0524 (10)	0.0643 (11)	0.0019 (8)	−0.0046 (8)	0.0082 (8)
C14	0.0568 (10)	0.0627 (11)	0.0633 (11)	−0.0039 (8)	0.0073 (8)	0.0106 (8)
C15	0.0437 (8)	0.0583 (10)	0.0685 (11)	−0.0044 (7)	0.0026 (7)	0.0099 (8)
C16	0.0880 (15)	0.0769 (14)	0.0691 (13)	−0.0031 (12)	−0.0106 (11)	0.0008 (11)
C17	0.0694 (12)	0.0678 (12)	0.0572 (11)	−0.0050 (9)	−0.0026 (9)	0.0135 (9)

### Geometric parameters (Å, º)

**Table:**

O1—C9	1.220 (2)	C8—H8	0.9300
N1—C17	1.136 (3)	C9—C10	1.481 (3)
C1—C2	1.394 (3)	C10—C15	1.392 (3)
C1—C6	1.398 (3)	C10—C11	1.399 (2)
C1—C7	1.464 (3)	C11—C12	1.381 (3)
C2—C3	1.373 (3)	C11—H11	0.9300
C2—H2	0.9300	C12—C13	1.382 (3)
C3—C4	1.389 (3)	C12—H12	0.9300
C3—H3	0.9300	C13—C14	1.399 (3)
C4—C5	1.396 (3)	C13—C16	1.503 (3)
C4—C17	1.439 (3)	C14—C15	1.373 (3)
C5—C6	1.373 (3)	C14—H14	0.9300
C5—H5	0.9300	C15—H15	0.9300
C6—H6	0.9300	C16—H16A	0.9600
C7—C8	1.323 (3)	C16—H16B	0.9600
C7—H7	0.9300	C16—H16C	0.9600
C8—C9	1.489 (3)		
			
C2—C1—C6	118.38 (16)	C10—C9—C8	118.29 (15)
C2—C1—C7	118.95 (16)	C15—C10—C11	118.40 (17)
C6—C1—C7	122.64 (16)	C15—C10—C9	119.20 (16)
C3—C2—C1	121.61 (17)	C11—C10—C9	122.39 (16)
C3—C2—H2	119.2	C12—C11—C10	119.91 (17)
C1—C2—H2	119.2	C12—C11—H11	120.0
C2—C3—C4	119.20 (17)	C10—C11—H11	120.0
C2—C3—H3	120.4	C11—C12—C13	122.10 (17)
C4—C3—H3	120.4	C11—C12—H12	119.0
C3—C4—C5	120.28 (17)	C13—C12—H12	119.0
C3—C4—C17	120.01 (17)	C12—C13—C14	117.43 (17)
C5—C4—C17	119.69 (17)	C12—C13—C16	121.89 (18)
C6—C5—C4	119.81 (16)	C14—C13—C16	120.68 (18)
C6—C5—H5	120.1	C15—C14—C13	121.31 (17)
C4—C5—H5	120.1	C15—C14—H14	119.3
C5—C6—C1	120.71 (16)	C13—C14—H14	119.3
C5—C6—H6	119.6	C14—C15—C10	120.83 (16)
C1—C6—H6	119.6	C14—C15—H15	119.6
C8—C7—C1	127.33 (17)	C10—C15—H15	119.6
C8—C7—H7	116.3	C13—C16—H16A	109.5
C1—C7—H7	116.3	C13—C16—H16B	109.5
C7—C8—C9	121.20 (17)	H16A—C16—H16B	109.5
C7—C8—H8	119.4	C13—C16—H16C	109.5
C9—C8—H8	119.4	H16A—C16—H16C	109.5
O1—C9—C10	120.84 (17)	H16B—C16—H16C	109.5
O1—C9—C8	120.86 (17)	N1—C17—C4	178.8 (2)
			
C6—C1—C2—C3	0.0 (3)	O1—C9—C10—C15	22.7 (3)
C7—C1—C2—C3	−178.17 (16)	C8—C9—C10—C15	−156.09 (18)
C1—C2—C3—C4	−0.1 (3)	O1—C9—C10—C11	−156.2 (2)
C2—C3—C4—C5	0.4 (3)	C8—C9—C10—C11	25.0 (3)
C2—C3—C4—C17	−177.81 (17)	C15—C10—C11—C12	−0.4 (3)
C3—C4—C5—C6	−0.6 (3)	C9—C10—C11—C12	178.50 (16)
C17—C4—C5—C6	177.68 (16)	C10—C11—C12—C13	1.0 (3)
C4—C5—C6—C1	0.4 (3)	C11—C12—C13—C14	−0.2 (3)
C2—C1—C6—C5	−0.1 (3)	C11—C12—C13—C16	179.91 (18)
C7—C1—C6—C5	177.96 (16)	C12—C13—C14—C15	−1.2 (3)
C2—C1—C7—C8	−167.96 (18)	C16—C13—C14—C15	178.69 (19)
C6—C1—C7—C8	14.0 (3)	C13—C14—C15—C10	1.8 (3)
C1—C7—C8—C9	−176.11 (17)	C11—C10—C15—C14	−1.0 (3)
C7—C8—C9—O1	13.5 (3)	C9—C10—C15—C14	−179.91 (16)
C7—C8—C9—C10	−167.68 (18)		

### Hydrogen-bond geometry (Å, º)

*Cg*1 and *Cg*2 are the centroids of the C1–C6 and C10–C15 benzene rings, respectively.

**Table:**

*D*—H···*A*	*D*—H	H···*A*	*D*···*A*	*D*—H···*A*
C2—H2···*Cg*2^i^	0.93	2.98	3.645 (2)	129
C5—H5···*Cg*2^ii^	0.93	2.91	3.5929 (18)	132
C12—H12···*Cg*1^iii^	0.93	2.98	3.637 (2)	129
C15—H15···*Cg*1^iv^	0.93	2.99	3.604 (2)	125

Symmetry codes: (i) −*x*, −*y*+2, −*z*+1; (ii) −*x*+1, −*y*+1, −*z*+1; (iii) −*x*+1, −*y*+2, −*z*+1; (iv) −*x*, −*y*+1, −*z*+1.
